# From Cognition to Behavior: Associations of Workplace Health Culture and Workplace Health Promotion Performance With Personal Healthy Lifestyles

**DOI:** 10.3389/fpubh.2021.745846

**Published:** 2021-11-08

**Authors:** Yao-Tsung Chang, Feng-Jen Tsai, Ching-Ying Yeh, Ruey-Yu Chen

**Affiliations:** ^1^School of Public Health, Taipei Medical University, Taipei, Taiwan; ^2^Ph.D. Program in Global Health and Health Security, and Master Program in Global Health and Development, College of Public Health, Taipei Medical University, Taipei, Taiwan; ^3^Taipei Medical University, Graduate Institute of Health and Biotechnology Law, Taipei, Taiwan

**Keywords:** workplace health culture, workplace health promotion, healthy lifestyle, workplace health culture scale, health promotion performance

## Abstract

**Introduction:** The aim of this study was to explore associations of workplace health culture and workplace health promotion (WHP) performance with employees' healthy lifestyles and health statuses.

**Methods:** In total, 27 enterprises and 1,732 participants were recruited for a cross-sectional designed survey. At the group level, Workplace Health Scorecard was used to measure WHP performance, and it was filled out by the WHP representative at each workplace. At the personal level, a personal questionnaire was used to measure workplace health culture, healthy lifestyles, and health statuses. A hierarchical linear model analysis was used to assess correlations between these variables.

**Results:** Workplace health culture was significantly related to WHP performance, healthy lifestyles, and health statuses. In particular, the peer support domain was greatly related to healthy behaviors like physical activity (*β* = 0.596, *p* < 0.001), vegetable consumption (*β* = 0.291, *p* < 0.001) and fruit consumption (*β* = 0.285, *p* < 0.05), and it may illustrate the importance of establishing peer support to promote healthy behaviors.

**Conclusions:** WHP performance was significantly related to workplace health culture especially health policies, health climate, and peer and supervisor support. Hence, building a good workplace health culture should be taken seriously, and more studies exploring associations of health culture and WHP performance with employees' health are needed.

## Introduction

It has been over 30 years since the World Health Organization (WHO) promoted workplace health promotion (WHP) (1, 2). During the past three decades, thousands of studies on WHP have been published worldwide, targeting workplace safety, employee healthy lifestyles, reducing health and medical costs, improving health productivity, etc. and trying to make health promotion become a part of daily life. However, although most researchers reported that WHP programs have positive economic effectiveness (3–5), almost all WHP promoters stuck to a core issue: How to improve the participation rate of WHP. Regardless of how comprehensive and accessible a program is, a lack of employee involvement brings about no or insufficient benefits. Therefore, finding ways to increase participation rates, reduce barriers to participation, and increase the willingness to participate has finally become a hot research topic in recent years.

Taiwan has been promoting WHP for more than 20 years. The WHP certification program promoted by the government has provided free workplace health promotion counseling and a certification system since 2007 (6). Up to the present, over 24,000 workplaces have obtained certifications (7). The government referred to the models and tools of the WHO and the US Centers for Disease Control (CDC) (8–11) and appropriately modified and localized that information to make it suitable for implementation in Taiwan while adhering to the issue of improving the participation rate of WHP programs at the same time. Hence, we realized the potential to research on workplace health culture.

Culture is a concept related to employees' attitudes, behaviors, and norms, and it affects specific behaviors (12, 13). The culture of WHP, or “workplace health culture”, is defined by considering norms, shared values, the health climate/morale, leadership support, peer support, and touch points (14). A culture of health reflects the attitudes and perceptions of the health of a company's employees, and these attitudes and cognition affect healthy behaviors. Regardless of which theory of healthy behavior one considers, behavior is driven by attitudes and cognition (15–17). Therefore, before trying to improve participation rates of WHP programs, it is more important to explore the workplace health culture. Although workplace health culture is not a novel topic, it seems that there have been few publications in the past two decades.

Since we previously developed a Workplace Health Culture Scale (WHCS) for Taiwan (18), it is time to explore deeper into the health culture. This study aimed to explore associations of workplace health culture and WHP performance with employees' healthy lifestyles and health statuses. Based on this study, we wanted to learn more about the factors that could increase participation rates in WHP programs.

## Materials and Methods

This study was a cross-sectional study approved by the Institutional Review Board (IRB) of Taipei Medical University (no. N201903113). In this study, we recruited enterprises of different sizes and from different industry categories to ensure sufficient generalizability.

### Study Population and Features

We recruited participating companies from Taiwan's WHP certification program database to ensure that the recruited companies have sufficient knowledge of WHP to fill in the checklists and questionnaires in this survey. Since the certification has three different tiers according to different levels of WHP, there is a clear difference in the effectiveness of WHP between each level. We recruited companies that participated in the certification program in 2018 and collected date between May 2019 and June 2021. In 2018, a total of 885 companies in northern Taiwan renewed or received the certification, and among them data were collected from 27 enterprises, including 11 manufacturing establishments, four professional scientific enterprises, three publishing firms, three financial and insurance organizations, two utility companies, one wholesale and retail trade enterprise, one transportation and storage firm, one human health company, and one public administration and defense contractor. A health promotion representative from each company was assigned to recruit volunteers to fill out the questionnaire and be responsible for answering the Taiwan workplace health scorecard. Most of the representatives were nurses, occupational safety and hygiene officers, or human resources personnel who were familiar with issues relating to health promotion and employees' health conditions. Since the work style and workplace characteristics of each company are different, the health promotion representative distributed and collected questionnaires in the most suitable way for the company, such as recruiting respondents through internal company letters, health promotion activities, health seminars, etc. All full-time and part-time adult company employees were eligible to participate.

### Sample Size

The sample size of each enterprise was decided by the company size. According to the sampling standards suggested by Glenn (19), the sample size for ±10% precision levels where confidence level is 95% and *p* = 0.05 for companies with 100–150 people, it is recommended that the number of samples should be about 50 people. For companies with more than 1,000 people, it is recommended that the number of samples should be about 100 people. If it is a small business with fewer than 100 people, it is encouraged to use the census as much as possible to increase its representativeness. In addition, according to the recommendations of Comrey and Lee (20), since this study would adopt a multi-level analysis, there should be more than 1,000 samples. Hence, in this study, we suggested that small-size companies (fewer than 150 employees) complete as many as 50 forms and medium- and large-size companies complete 100 forms.

### Measures

Workplace health culture was measured by the WHCS (18), which contained 25 items in six domains of health policy, health climate, peer support, supervisor support and role modeling, personal values, and common values. We developed this Traditional Chinese questionnaire in 2019. WHCS has good construct validity and can explain about 69% of the variation, and the Cronbach's α of each domain is between 0.804 and 0.919. Every item began with “I think…” or “My colleagues and I feel that…” to reflect employees' attitudes and feelings, and items were rated on a five-point Likert scale, ranging from “strongly agree” (5 points) to “strongly disagree” (1 point). We calculated scores for each domain and a total score. The items and total scores of the six domains were: Health policy: 3 items, 3–15 points; health climate:7 items, 7–35 points; peer support: 3 items, 3–15 points; supervisor support: 4 items, 4–20 points; personal value: 3 items, 3–15 points; and common value: 5 items, 5–25 points.

WHP performance was measured by the “Taiwan Workplace Health Scorecard” (21), and it was filled out by the person responsible for promoting WHP in each workplace. This tool was designed by referring to the US CDC's worksite health scorecard for a tool suitable to Taiwan's current environment and regulations of the WHP system, and it contained 46 items in five domains of health policy and planning (seven items, 15 points), workplace health needs assessments (four items, 10 points), health promotion activities (23 items, 50 points), healthy work environment (nine items, 20 points), and enterprise community involvement (three items, 5 points). All items were in a Yes/No format. Each item had a weighted point value (1–3 points) according to its importance. Overall and domain scores were summed on the items that received a “Yes” response, and “No” responses were given 0 points.

Healthy lifestyle variables were comprised of physical activity, vegetable consumption, fruit consumption, and regular weight measurement. Physical activity was measured with the Godin leisure-time physical activity scale (22, 23), which marks the number of days in a week a subject does vigorous (9 points), medium (5 points), and light (3 points) physical activities. After weighting and summing up each level of physical activity, it defines an active person as one with a total of ≥24 points, a moderately active person as one with 14–23 points, and an insufficiently active person as one with ≤13 points. Both vegetable and fruit consumption levels were measured by a single item, which evaluated the number of servings per day consumed in the past week. According to WHO's recommendations (24), we set the sufficient vegetables and fruits intake to at least three servings of vegetables and two servings of fruits a day. Regular weight measurement behavior was also measured by a single item to evaluate the frequency of weighing oneself.

Health status variables consisted of self-rated health, mental health, and the number of chronic diseases. Self-rated health was measured by a six-point item, ranging from “very good” (6 point) to “very bad” (1 point). Mental health was measured by the Brief Symptom Rating Scale Short Version (BSRS-5), which has good reliability and validity (25). It is a five-item, self-rated questionnaire, with each item ranging from 4 (“extremely”) to 0 (“not at all”). Total scores of <5 points are considered “normal mental health”, 6–9 points with a “mild mood disorder”, 10–14 points as a “moderate mood disorder”, and >14 might as a “severe mood disorder”. Chronic diseases were measured by a multiple-choice question, and the total number was calculated.

Sociodemographic variables included group- and personal-level items. Group-level items included the enterprise size and industry category. Personal-level items included gender, age, educational level, and position. We classified managers above the first-line supervisor as supervisors, and other employees, including general staff, researchers, professional services, etc., were classified as general staff.

### Statistical Analysis

An analysis of variance (ANOVA) test and Chi-squared test were employed to assess differences in sociodemographic factors, healthy lifestyles, health statuses, workplace health culture, and WHP performances among different-sized enterprises at the baseline (Tables 1, 2). Pearson correlations were used to assess correlations between workplace health culture at the personal level and WHP performance (Table 3). Before the model analysis, we assessed simple correlations among sociodemographic factors, healthy lifestyles, health statuses, and personal-level workplace health culture to explore potential confounding variables. Finally, a hierarchical linear model analysis was used to assess correlations of personal-level workplace health culture (dependent variables) with healthy lifestyles and health statuses (independent variables), setting the company as the group-level adjusted variable and adjusting for gender, age, educational level, position, and enterprise size. The reference group set in the hierarchical linear model included those with insufficient physical activity, insufficient vegetable and fruit consumption, weight measurement less than weekly, and with a normal weight and normal mental health. All analyses were performed according to the intention-to-treat principle, and all tests were analyzed at a 95% significance level (*p* < 0.05). Analyses were conducted using PASW 22.0 software for Windows (SPSS, Chicago, IL, USA).

**Table 1:** Basic demographic characteristics, healthy lifestyles, and health status information.

	**Enterprise size^a^**			***p*** **-value**
	**Small (*N* = 3, *n* = 131)**	**Medium (*N* = 18, *n* = 1,046)**	**Large (*N* = 6, *n* = 555)**	
**Demographic characteristic**, ***n*** **(%)**				
Gender				0.025^*^
Male	48 (36.6)	514 (49.2)	266 (48.0)	
Female	83 (63.4)	530 (50.8)	288 (52.0)	
Age (years)				0.074
18–29	13 (10.2)	168 (16.2)	93 (16.8)	
30–39	46 (35.9)	423 (40.7)	231 (41.8)	
40–49	51 (39.8)	289 (27.8)	142 (25.7)	
≥50	18 (14.1)	160 (15.4)	86 (15.6)	
Educational level				<0.001^**^
Senior high school or below	3 (2.3)	123 (11.9)	34 (6.1)	
University	91 (70.5)	553 (53.5)	257 (46.6)	
Master's degree or above	35 (27.1)	358 (34.6)	261 (47.3)	
Job position				0.001^**^
Supervisor	23 (18.3)	100 (9.7)	80 (14.8)	
General staff	103 (81.7)	935 (90.3)	460 (85.2)	
**Healthy lifestyle and health status**				
Physical activity				<0.001^**^
Insufficiently active	75 (57.7)	542 (52.2)	235 (42.8)	
Moderately active	31 (23.8)	205 (19.7)	114 (20.8)	
Active	24 (18.5)	291 (28.0)	200 (36.4)	
Vegetable consumption				0.136
Insufficiently	44 (33.8)	344 (33.0)	211 (38.0)	
Sufficient	86 (66.2)	697 (67.0)	344 (62.0)	
Fruit consumption				0.228
Insufficiently	50 (38.5)	337 (32.3)	196 (35.4)	
Sufficient	80 (61.5)	707 (67.7)	358 (64.6)	
Regular weight measurement				0.020^*^
Almost everyday	7 (5.3)	57 (5.5)	50 (9.1)	
Weekly	58 (44.3)	373 (35.8)	192 (34.8)	
Monthly or less	66 (50.4)	612 (58.7)	310 (56.2)	
Mental health				0.600
Normal	113 (86.3)	875 (83.7)	469 (84.5)	
Light pressure	16 (12.2)	115 (11.0)	63 (11.4)	
Medium pressure	2 (1.5)	47 (4.5)	19 (3.4)	
High pressure	0 (0.0)	9 (0.9)	4 (0.7)	
Body-mass index				0.137
Underweight	4 (3.1)	42 (4.1)	11 (2.0)	
Normal weight	72 (55.4)	538 (53.1)	297 (55.2)	
Overweight	50 (38.5)	350 (34.6)	192 (35.7)	
Obese	4 (3.1)	83 (8.2)	38 (7.1)	
Self-rated health (mean ± SD)	3.35 ± 0.84	3.38 ± 0.91	3.46 ± 0.94	0.166

a *Small size: <150 employees, medium size: 150–999 employees, large size: ≥1,000 employees. n, number of participants; N, number of enterprises*.

* *0.01 < p < 0.05*,

** *p < 0.01*.

*SD, standard deviation*.

**Table 2:** Characteristic of workplace health culture and workplace health promotion (WHP) performance.

	**Enterprise size (Mean** **±** **SD)**			***p-*** **value**
	**Small (*N* = 3, *n* = 131)**	**Medium (*N* = 18, *n* = 1,046)**	**Large (*N* = 6, *n* = 555)**	
**Workplace health culture^a^**				
Health policy	10.48 ± 3.61	10.87 ± 3.56	11.46 ± 3.04	0.001^**^
Health climate	23.55 ± 5.48	24.66 ± 5.39	25.63 ± 4.79	<0.001^**^
Peer support	11.21 ± 2.20	11.20 ± 2.16	11.58 ± 2.12	0.004^**^
Supervisor support	13.84 ± 2.82	14.40 ± 2.95	14.63 ± 2.94	0.018^*^
Personal values	11.24 ± 1.86	11.00 ± 2.10	11.30 ± 1.98	0.015^*^
Common values	21.03 ± 2.72	20.35 ± 2.95	21.10 ± 2.79	<0.001^**^
**WHP performance**				
Health policies and plans	11.33 ± 6.35	14.17 ± 1.54	13.33 ± 1.86	0.187
Health needs assessments	8.33 ± 2.89	9.78 ± 0.73	10.00 ± 0.00	0.076
Health promotion activities	39.33 ± 12.90	42.56 ± 7.21	40.50 ± 3.27	0.616
Healthy work environment	20.00 ± 0.00	19.61 ± 0.92	18.00 ± 1.67	0.100
Community involvements	3.33 ± 2.31	3.50 ± 1.79	3.33 ± 1.97	0.189

a *The total scores of each domains are: Health policy: 3 items, 3–15 points; health climate: 7 items, 7–35 points; peer support: 3 items, 3–15 points; supervisor support: 4 items, 4–20 points; personal value: 3 items, 3–15 points; and common value: 5 items, 5–25 points*.

* *0.01 < p < 0.05*,

** *p < 0.01*.

*SD, standard deviation*.

**Table 3:** Correlations of personal health culture cognition with company workplace health promotion (WHP) performance.

**WHP performance**	**Workplace health culture**					
	**Health policies**	**Health climate**	**Peer support**	**Supervisor support**	**Personal values**	**Common values**
Health policies and plans	0.118^**^	0.158^**^	0.093^**^	0.083^**^	0.016	0.022
Health needs assessments	0.111^**^	0.182^**^	0.080^**^	0.080^**^	0.033	0.014
Health promotion activities	0.107^**^	0.128^**^	0.047	0.055^*^	0.016	−0.015
Healthy work environment	0.023	0.004	−0.020	0.029	−0.004	−0.022
Community involvement	0.151^**^	0.161^**^	0.146^**^	0.132^**^	0.108^**^	0.089^**^

* *p < 0.05*,

** *p < 0.01*.

## Results

Between May 2019 and June 2021, 27 enterprises and eventually a total of 1,732 subjects were enrolled in the study, including three small enterprises, 18 medium enterprises, and six large enterprises. The demographic characteristics of the study group are listed in Table 1. Of the 27 enterprises, small enterprises had significantly more female employees, medium enterprises had significantly more general staff, and large enterprises had the most employees with a master's degree or above. Most health behaviors exhibited no significant differences among enterprise sizes except for physical activity and regular weight measurement. Large enterprises had the lowest percentage of employees who were insufficiently active and the highest percentage of employee who were active. Of 1,732 participants, 52.0% were female, the mean age was 39.3 (standard deviation (SD) = 9.55) years, 90.3% had a bachelor's degree or higher, 88.1% were general staff, the mean body-mass index (BMI) was 24.0 (SD = 4.09) kg/m^2^, and 42.7% were overweight or obese. As to healthy lifestyles, 49.6% of participants were insufficiently active, 66.3% consumed insufficient fruit, 65.3% consumed insufficient vegetables every day, and 17.2% were smokers. As to the health statuses, 84.1% had good mental health, while 50.3% rated their health status as normal and 38.2% as good.

All indicators of the workplace health culture and WHP performance were significant among different enterprise sizes (Table 2). Large enterprises had the best health culture, but WHP performances did not significantly differ among different enterprise sizes.

Correlations of personal level workplace health culture and WHP performance are listed in Table 3. A healthy work environment was not significantly correlated with any of the workplace health culture domains, and community involvement was significantly related to all health culture domains. In addition, personal values and common values were not significantly related with almost any WHP performance domains except community involvement.

Results of the hierarchical linear model analysis of workplace health culture with healthy lifestyles and health statuses are listed in Table 4. People who had better physical activity, vegetable consumption, and fruit consumption and measured their weight daily or weekly felt that they had significantly better peer support. With better physical activity habits, the participants had better personal values and common values of health. If they measured their weight every day or at least once per week, they felt that they had better peer support, supervisor support, and common values of health. People who had better self-rated health believed that they had a significantly better workplace health culture. In addition, the participants with fewer chronic diseases felt that they had significantly better health policies and health climate support. Also, people who had better mental health thought that they had a significantly better workplace health culture except for peer support. However, it seems that the BMI had no significant correlation with the workplace health culture.

**Table 4:** Hierarchical linear model analysis: associations of workplace health culture with healthy lifestyles and health statuses, after adjusting for gender, age, educational level, position, and business size.

**Variable**	**Workplace health culture** **β** **value**					
	**Health policies**	**Health climate**	**Peer support**	**Supervisor support**	**Personal values**	**Common values**
**Healthy lifestyle**						
Physical activity	0.293^#^	0.321	0.596^**^	0.078	0.306^**^	0.416^**^
Vegetable consumption	−0.224	0.015	0.291^**^	0.074	−0.103	0.035
Fruit consumption	0.221	0.231	0.285^*^	0.209	0.138	0.171
Frequent weight measurement	0.312^#^	0.538^*^	0.409^**^	0.433^**^	0.158	0.334^*^
**Health status**						
Self-rated health	0.346^**^	0.873^**^	0.456^**^	0.479^**^	0.286^**^	0.424^**^
Number of chronic diseases	−0.280^**^	−0.415^**^	−0.042	−0.123	−0.065	−0.029
**Body-mass index (kg/m** ^**2**^ **)**						
<18	−0.180	−1.304^#^	−0.090	−0.469	0.226	−0.441
≥24	0.208	0.216	−0.017	0.190	0.003	0.272^#^
Mental health	−0.658^**^	−2.086^**^	−0.230	−0.875^**^	−0.277^*^	−0.280

# *0.05 < p < 0.10*,

* *0.01 < p < 0.05*,

** *p < 0.01*.

## Discussion

In this study, we found that WHP performance was significantly related with workplace health culture especially health policies, health climate, and peer and supervisor support, but was less related to personal and common values of health. In addition, self-rated health and mental health were significantly related to the health culture, and people who had better physical activity habits felt that they had a better workplace health culture.

The significant correlation between health culture and WHP performance means that the more a company invests in WHP, especially in the domains of health policies, health needs assessments, and health behavior promotion, the better their employees will feel about health culture. Employees can really feel and agree with the company's efforts to promote WHP though there is not much research on health culture as seen in the research on safety culture (26, 27). In addition, those enterprises with better community involvement also had significantly better health cultures, e.g., providing health promotion to employees' family members, affiliated companies, and people in the community.

The reason why there were no significant correlations between a healthy work environment and any of the workplace health culture domains may have been because this sub-scale focused on the safety environment rather than healthy lifestyles, and these items were related to the basic safety environment which most workplaces can meet. Some studies also found that it is not easy to change the health behaviors of employees by merely improving the health environment without promoting healthy lifestyles (28, 29). This does not mean that improving the health environment is not important, but designing and promoting workplace health policies, promoting health needs assessments, and actual health promotion actions can allow employees to experience the health culture more directly. After all, creating a supportive environment and making more-convenient, accessible, and healthier choices will also benefit healthy lifestyle building (30).

An interesting point is that in this study we found that there is almost no significant correlation between the scores of personal value and common value and WHP performance. This may indicate that basic workplace health promotion is not easy to change employees' health values related to WHP. In past studies, health values were mainly discussed on issues related to health beliefs, and health beliefs are the mediator of health culture and health behaviors (31). Although personal health beliefs are indeed related to personal health behaviors, there have been few discussions on beliefs related to participation in workplace health promotion in the past. This may mean that most studies did not regard participation in workplace health promotion as a “behavior”. Although in recent years there have been more and more health coaching studies in the workplace region to improve the employees' healthy lifestyle and the WHP participation (32, 33), whether health coaching can improve employees' personal and common values of WHP still needs more studies.

It is not surprising that large-sized companies had significantly better health cultures in this study. Many studies found that smaller companies are less likely to fund WHP implementation due to fewer resources and experience than larger companies (34, 35), and the rate of implementing WHP in small companies was indeed lower than that in large companies. However, the enterprise size might not be the main factor in the WHP performance, since smaller companies can more easily achieve higher participant rates than large companies (36), and smaller companies also have relatively simple company structures which might make it easier for them to promote comprehensive WHP. This might explain why the WHP performance was not significant among enterprise sizes in our study.

In this study, we found those people who rated having a higher peer support score had significantly healthier lifestyles, and those with higher self-rated health also felt that they had a better health culture. In addition, people with better health e.g., fewer chronic diseases and better mental health, felt that they had better health policies and a healthy climate. Interestingly, it seemed that most health culture domains did not affect a healthy diet except for peer support, but one study indeed indicated that peer support and role models can help promote healthy eating (37). It also might mean that peer support is the most important cultural factor in improving personal healthy lifestyles. However, physical activity was also related to personal values and common values. One of the reasons why there were different results from a healthy diet may be because Taiwan has worked hard to promote physical activity in the workplace in recent years, providing a considerable number of resources to promote it in the workplace, or perhaps a healthy diet is more irrelevant to personal and common values. As to the correlation between chronic diseases and health culture, a possible explanation for only health policies and a healthy climate being significantly related to it is that interpersonal health support and values might have more direct correlations with a healthy lifestyle and an indirect linkage to the health status, but this needs to be verified by future studies.

Finally, the assumption and some findings from other studies that health promotion cognitive/attitudes/values affect motivations for healthy behaviors and actual healthy behaviors, and thus affect the health status may be correct (31, 38). In the past, there were many studies which found that supervisor support could increase employees' participation in WHP (39, 40), and the participation rate greatly determines the effectiveness of WHP implementation (41). In fact, “participation in health promotion” is a kind of behavior, and it is inevitably affected by motivation. Many studies have explored ways to increase participants' motivation and participation rates in WHP programs or found out the reason why employees do not participate in such programs (30, 42, 43). For example, employers can provide participation rewards, shape a health-promoting environment, and provide healthy working conditions, which will be reflected in the domain of health policies and supervisor support of culture. Peer pressure and support are also important motivations for participation (44, 45), which will be reflected in the domain of peer support and common values of culture.

The main strength of this paper is that it is the first study conducted to examine correlations of workplace health culture and WHP performance with healthy lifestyles and health statuses. In addition, even though WHP has been promoted for quite a long time, so far, there have been few studies with large-scale investigations attempting to explore the correlation between WHP in the work environment, personal health behavior, and personal cognition. This study has a large sample size involving 27 enterprises, making it possible to use multi-level analysis to explore these relationships more comprehensively. Moreover, the scarcity of research related to workplace health culture also makes this research more important. However, there remain some limitations. First, the cross-sectional design did not allow us to draw causal relationships among these variables. Considering the assumptions that we can improve employees' motivation to engage in healthier behaviors by implementing WHP programs, this should involve a large, long-term, rigorous longitudinal test. Second, the workplaces which participated in this experiment may have had better WHP performances and a greater willingness to implement it. This might explain why the most WHP performance indicators had no significant difference among enterprise sizes in our study. Those workplaces that were willing to participate in this research may have had a higher willingness to promote WHP in the first place. At the same time, some samples in this study were collected during the COVID-19 pandemic, and pandemic prevention policies and workplace restrictions may affect the willingness to participate in the investigation. In addition, although some studies have pointed out that strict prevention policies during the COVID-19 pandemic may affect work efficiency and quality of life (46, 47), it caused little impact on the validity of this study due to the fact that Taiwan is only slightly affected by the COVID-19 pandemic. Hence, to heighten generalizability and representativeness of similar research in the future, diversified industries or companies should be incorporated.

Here are some suggestions for future work on WHP and studies according to our study results. First, actual health policy making and communication with employees, regular health needs assessments, and health promotion activities are worth implementing because these actions can improve the health culture except values. Considering that peer support is the most influential factor in a healthy lifestyle, implementing WHP should indeed help improve employee health through promoting healthy lifestyles. Second, building stronger peer support such as encouraging the building of healthy communities, organizing team competitions, and providing group workshops or group coaching to provide social support may improve healthy lifestyles more efficiently. And finally, it is important to provide resources to increase the willingness of small enterprises to invest in WHP. Our study results showed that small enterprises can also implement comprehensive and effective WHP, but the number of small enterprises that can do this is indeed far fewer than that of medium and large companies. Of course, it is also necessary to conduct more-comprehensive research on health culture, WHP performance, and employee health.

## Data Availability Statement

The raw data supporting the conclusions of this article will be made available by the authors, without undue reservation.

## Ethics Statement

The studies involving human participants were reviewed and approved by Institutional Review Board of Taipei Medical University. Written informed consent for participation was not required for this study in accordance with the national legislation and the institutional requirements.

## Author Contributions

Y-TC and R-YC oversaw all aspects of the study. Y-TC contributed to study design, data collection, data analysis, interpretation of results, and wrote the first draft of the manuscript. F-JT contributed to study design, data collection, and data analysis. C-YY contributed to study design and data analysis. R-YC contributed to study design, data collection, interpretation of results, manuscript writing, and compiled edits from other authors. All authors had full access to all the data in the study and had final responsibility for the decision to submit for publication.

## Conflict of Interest

The authors declare that the research was conducted in the absence of any commercial or financial relationships that could be construed as a potential conflict of interest.

## Publisher's Note

All claims expressed in this article are solely those of the authors and do not necessarily represent those of their affiliated organizations, or those of the publisher, the editors and the reviewers. Any product that may be evaluated in this article, or claim that may be made by its manufacturer, is not guaranteed or endorsed by the publisher.

## Abbreviations

WHP: Workplace Health Promotion
WHCS: Workplace Health Culture Scale
IRB: Institutional Review Board
SD: Standard deviation.
